# Identification of a hemorrhagic determinant in *Clostridioides difficile* TcdA and *Paeniclostridium sordellii* TcsH

**DOI:** 10.1128/spectrum.00354-24

**Published:** 2024-05-06

**Authors:** Yangling Zheng, Qi Yang, Jianhua Luo, Yuanyuan Zhang, Xingxing Li, Liuqing He, Chao Ma, Liang Tao

**Affiliations:** 1 College of Life Sciences, Zhejiang University, Hangzhou, Zhejiang, China; 2 Center for Infectious Disease Research, Westlake Laboratory of Life Sciences and Biomedicine, Hangzhou, Zhejiang, China; 3 Research Center for Industries of the Future and Key Laboratory of Multi-omics in Infection and Immunity of Zhejiang Province, School of Medicine, School of Life Sciences, Westlake University, Hangzhou, Zhejiang, China; 4 Institute of Basic Medical Sciences, Westlake Institute for Advanced Study, Hangzhou, Zhejiang, China; Cinvestav-IPN, Mexico City, Mexico

**Keywords:** *Clostridium difficile*, *Paeniclostridium sordellii*, TcdA, TcsH, bacterial toxin

## Abstract

*Paeniclostridium sordellii* hemorrhagic toxin (TcsH) and *Clostridioides difficile* toxin A (TcdA) are two major members of the large clostridial toxin (LCT) family. These two toxins share ~87% similarity and are known to cause severe hemorrhagic pathology in animals. Yet, the pathogenesis of their hemorrhagic toxicity has been mysterious for decades. Here, we examined the liver injury after systemic exposure to different LCTs and found that only TcsH and TcdA induce overt hepatic hemorrhage. By investigating the chimeric and truncated toxins, we demonstrated that the enzymatic domain of TcsH alone is not sufficient to determine its potent hepatic hemorrhagic toxicity in mice. Likewise, the combined repetitive oligopeptide (CROP) domain of TcsH/TcdA alone also failed to explain their strong hemorrhagic activity in mice. Lastly, we showed that disrupting the first two short repeats of CROPs in TcsH and TcdA impaired hemorrhagic toxicity without causing overt changes in cytotoxicity and lethality. These findings lead to a deeper understanding of toxin-induced hemorrhage and the pathogenesis of LCTs and could be insightful in developing therapeutic avenues against clostridial infections.

**IMPORTANCE:**

*Paeniclostridium sordellii* and *Clostridioides difficile* infections often cause hemorrhage in the affected tissues and organs, which is mainly attributed to their hemorrhagic toxins, TcsH and TcdA. In this study, we demonstrate that TcsH and TcdA, but not other related toxins. including *Clostridioides difficile* toxin B and TcsL, induce severe hepatic hemorrhage in mice. We further determine that a small region in TcsH and TcdA is critical for the hemorrhagic toxicity but not cytotoxicity or lethality of these toxins. Based on these results, we propose that the hemorrhagic toxicity of TcsH and TcdA is due to an uncharacterized mechanism, such as the presence of an unknown receptor, and future studies to identify the interactive host factors are warranted.

## INTRODUCTION

Clostridia are a group of Gram-positive anaerobic bacteria that commonly reside in the environment. Some clostridial species, such as *Clostridioides difficile* and *Paeniclostridium sordellii*, are pathogenic to humans and other animals owing to the production of large clostridial toxins (LCTs) (1). Their infections lead to a broad spectrum of symptoms such as myonecrosis, gangrene, peritonitis, diarrhea, hemorrhagic enteritis, colitis, sepsis, and even death (2–4).

LCTs are a family of highly potent exotoxins, which consist of *Clostridioides difficile* toxin A (TcdA) and *Clostridioides difficile* toxin B (TcdB), *Paeniclostridium sordellii* hemorrhagic toxin (TcsH) and *Paeniclostridium sordellii* lethal toxin (TcsL), *Clostridium novyi* alpha-toxin (Tcnα), and *Clostridium perfringens* large cytotoxin (TpeL) (5). These toxins are usually composed of four structural domains: an N-terminal glucosyltransferase domain (GTD) that glycosylates and inactivates Rho/Ras family GTPases, a cysteine protease domain (CPD) that releases the GTD in the cytosol via autocleavage, a combined translocation and receptor-binding domain (DRBD), and followed with a combined repetitive oligopeptides (CROPs) (6–8). TcsH and TcdA are closely related within the LCT family, with a sequence similarity of ~82.8%. These two toxins also contain the longest CROP domains among LCTs (5). The TcsH CROP domain is composed of 29 short repeats (SRs) of 20–24 amino acids interspersed with 6 long repeats (LRs) of 31 amino acids (9), while the CROP domain of TcdA consists of 32 SRs and 8 LRs.

Among LCTs, TcsH is well known for its distinctive hemorrhagic toxicity and is named after it (H stands for hemorrhagic) (10, 11). The hemorrhagic toxicity of TcsH was first described in 1983 using the rabbit intestinal ligation experiment (12). Later studies demonstrated that both TcsH and TcdA caused strong hemorrhage in different animal models (11, 13–16). Clinically, *P. sordellii* and *C. difficile* infections commonly cause signs and symptoms associated with hemorrhage, such as bloody diarrhea, hemorrhagic enteritis, hypotension, and sepsis (1), likely due to the hemorrhagic toxicity of TcdA and TcsH. However, the precise underlying mechanisms of this toxin-induced hemorrhagic pathology have remained elusive for many years. Here, we examined the liver injury following systemic exposure to different LCTs. TcsH and TcdA, but not other LCTs, led to overt hepatic hemorrhage at their lethal doses. This provides us with a feasible and reliable model to investigate the hemorrhagic toxicity of LCTs in mice. We then tested several chimeras of TcdB and TcsH or TcdA, but none of them caused a hepatic hemorrhage in mice, indicating that the TcsH/TcdA GTD or CROP domain alone appears to be insufficient for specifying the hemorrhagic toxicity. By studying the truncated TcsH, we showed that whereas the entire CROP domain is essential, the C-half CROPs are dispensable for the hemorrhagic toxicity of TcsH. Moreover, disrupting the first two SRs of CROPs specifically impairs the hemorrhagic toxicity of both TcsH and TcdA, indicating this small region serves as a shared hemorrhagic determinant. Importantly, our study demonstrates that the hemorrhagic toxicity can be separated from the cytotoxicity or lethal toxicity in TcsH and TcdA, suggesting the presence of the yet unknown factors/signaling specific to the hemorrhagic pathology induced by these toxins.

## RESULTS

### TcsH- and TcdA-induced hepatic hemorrhage is specific among LCTs

**Fig 1:**
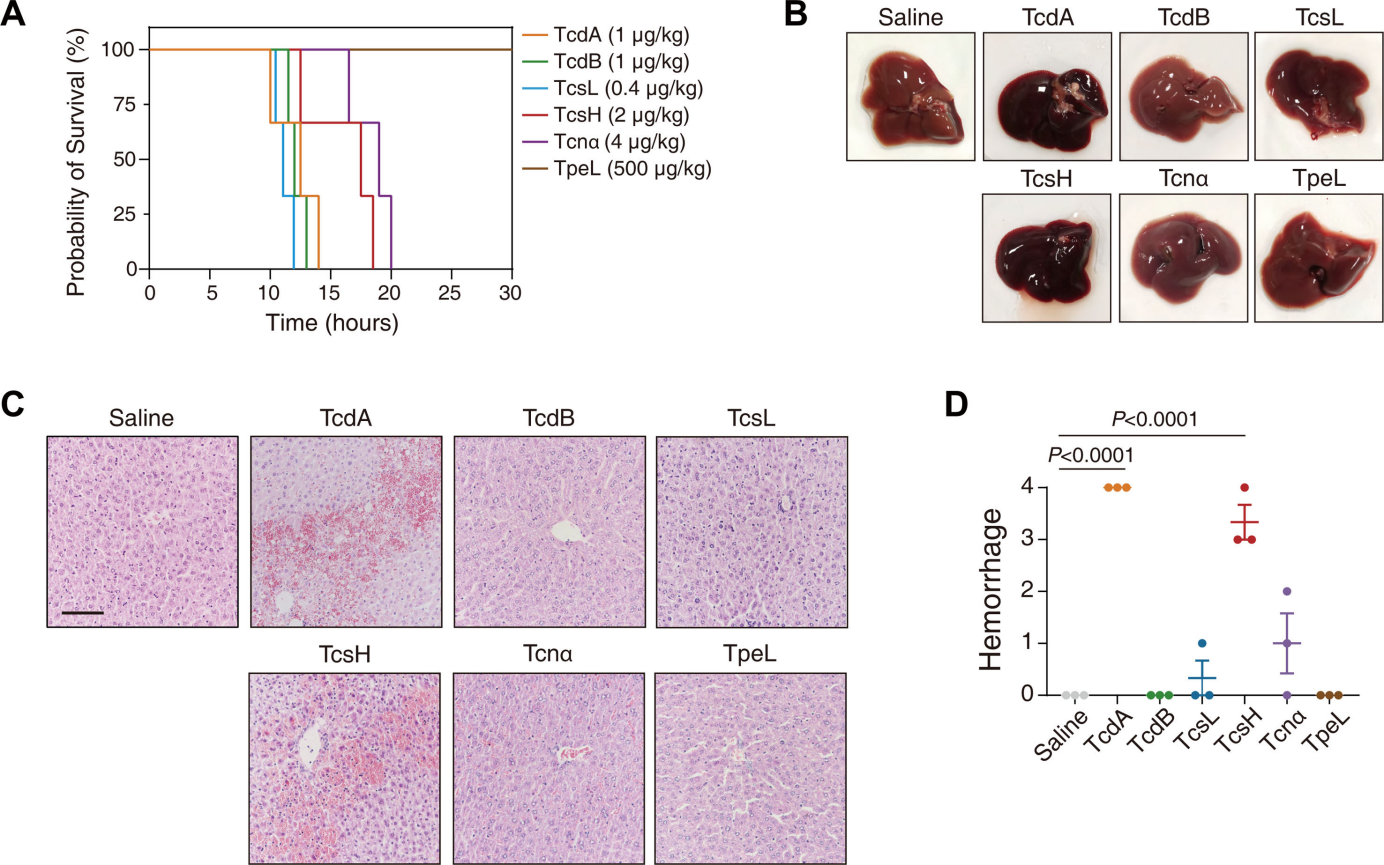
TcsH and TcdA-induced hepatic hemorrhage is specific among LCTs. (**A**) Mice were IP injected with 1-µg/kg TcdA, 1-µg/kg TcdB, 2-µg/kg TcsH, 0.4-µg/kg TcsL, 4-µg/kg Tcnα, 500-µg/kg TpeL, or saline. The Kaplan-Meier survival curves are shown. (**B**) The livers were dissected from the mice 8 hours post-injection with different LCTs. Representative pictures are shown. (**C**) Hematoxylin and eosin-stained liver sections from the mice 8 hours post-injection with different LCTs. Representative images are shown (scale bar, 100 µm). (**D**) The stained sections in panel **C** were analyzed, and histological scores for hemorrhage were assessed. Error bars indicate mean ± SEM, *n* = 3 mice per group, ordinary one-way analysis of variance.

In the previous study, we reported that intraperitoneally (IP) injected TcsH led to severe hepatic hemorrhage in mice (17). To demonstrate whether the TcsH-induced hemorrhagic pathology is specific among the LCT family, we respectively challenged mice with six LCTs, including TcsH, TcsL, TcdA, TcdB [TcdB1 (18), here and thereafter], Tcnα, and TpeL, by IP injection. Because LCTs may have varied lethal toxicities in mice, we chose the toxin dose that would kill mice within ~12 to 24 hours in the toxin challenge assays (this criterion is used thereafter). The only exception is TpeL, as all mice survived even when a very high dosage (500 µg/kg) was applied (Fig. 1A), which was consistent with the previous report (19).

The mice were injected with 2-µg/kg TcsH, TcdA, and Tcnα, 1-µg/kg TcdB, 0.4-µg/kg TcsL, or 500-µg/kg TpeL; euthanized 8 hours post-toxin injection; and dissected for pathological examination. The livers of mice challenged with TcsH or TcdA showed a reddish-black color, while others were reddish-brown (Fig. 1B). Histopathological analysis of the hematoxylin and eosin (H&E)-stained liver sections revealed massive extravasation of erythrocytes, in company with hepatocyte necrosis in the hepatic lobules, in the TcsH and TcdA groups. In contrast, TcdB, Tcnα, TcsL, and TpeL caused no overt hepatic hemorrhage (Fig. 1C and D). Thus, we consider the assessment of liver damage following systemic toxin exposure as a feasible mouse model to study the hemorrhagic toxicity of LCTs *in vivo*.

### The hemorrhagic toxicity is not determined by target protein specificity

**Fig 2:**
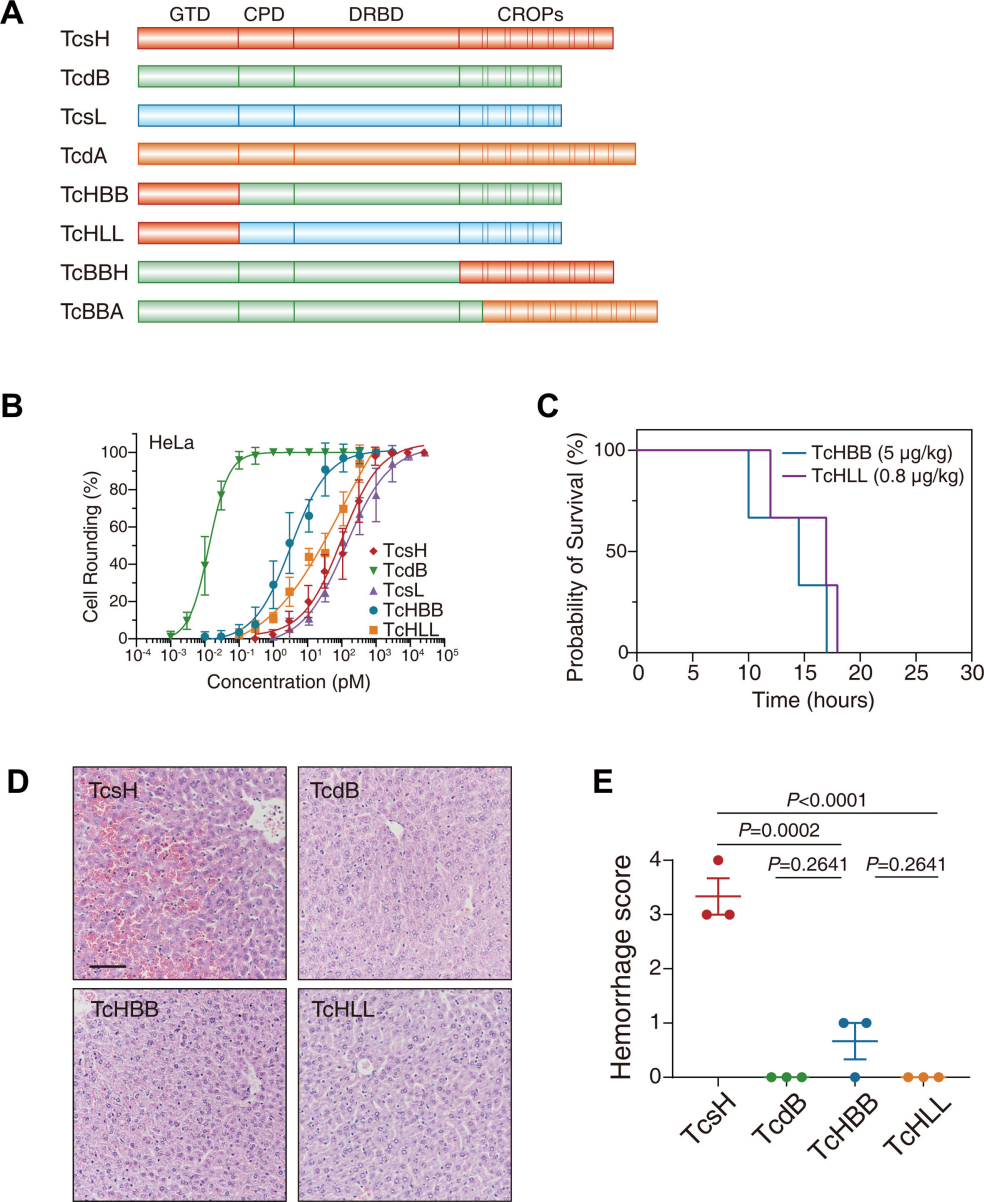
The hemorrhagic toxicity is not determined by target specificity. (**A**) Schematic drawing of designed chimeric toxins TcHBB, TcHLL, TcBBH, and TcBBA. (**B**) The sensitivities of HeLa WT cells toward TcsH, TcdB, TcsL, TcHBB, and TcHLL were measured using the cytopathic cell-rounding experiments. Error bars represent the mean ± SD, *n* = 6. (**C**) Kaplan-Meier curves show the survival of C57BL/6 WT mice intraperitoneally injected with 5-µg/kg TcHBB and 0.8-µg/kg TcHLL, respectively. (**D**) Mouse liver tissues were harvested 8 hours post-toxin injection and sectioned for H&E staining histopathology (scale bar, 100 µm.). (**E**) Histological scores for the hemorrhagic pathology in panel** D** were assessed. Error bars indicate mean ± SEM, *n* = 3 mice per group, ordinary one-way analysis of variance. CPD, cysteine protease domain; CROP, combined repetitive oligopeptide domain; DRBD, delivery and receptor-binding domain; GTD, glucosyltransferase domain; WT, wild type.

Albeit LCTs generally target Rho/Ras family small GTPase proteins, varied LCT members may prefer different protein targets (6, 20). For example, TcsL glucosylates Rac1 and H/N/K-Ras, while TcdB and TcsH mainly glucosylate RhoA/B/C, Rac1, and Cdc42 (21, 22). To investigate whether the target protein preference of TcsH determines its hemorrhagic toxicity, we created chimeric toxins (TcHBB and TcHLL) by combining residues 1–520 from TcsH and residues 522–2366 from TcdB or TcsL. These chimeras have an enzymatic domain of TcsH but other domains from either TcdB or TcsL (Fig. 2A).

Both TcHBB and TcHLL showed comparable cytotoxicity to TcsL and TcsH in the HeLa cells, suggesting they are functionally active (Fig. 2B). In the toxin challenge assay, 5-µg/kg TcHBB or 0.8-µg/kg TcHLL killed the mice in ~12 to 24 hours post-IP injection, indicating that these chimeric toxins are active (Fig. 2C; Fig. S1A). Neither TcHBB nor TcHLL caused overt hepatic hemorrhagic lesions (Fig. 2D and E), indicating that the difference in target preference among LCTs is unlikely a decisive factor in inducing hepatic hemorrhage *in vivo*.

### TcdB fused with TcsH or TcdA CROPs caused no hepatic hemorrhage

**Fig 3:**
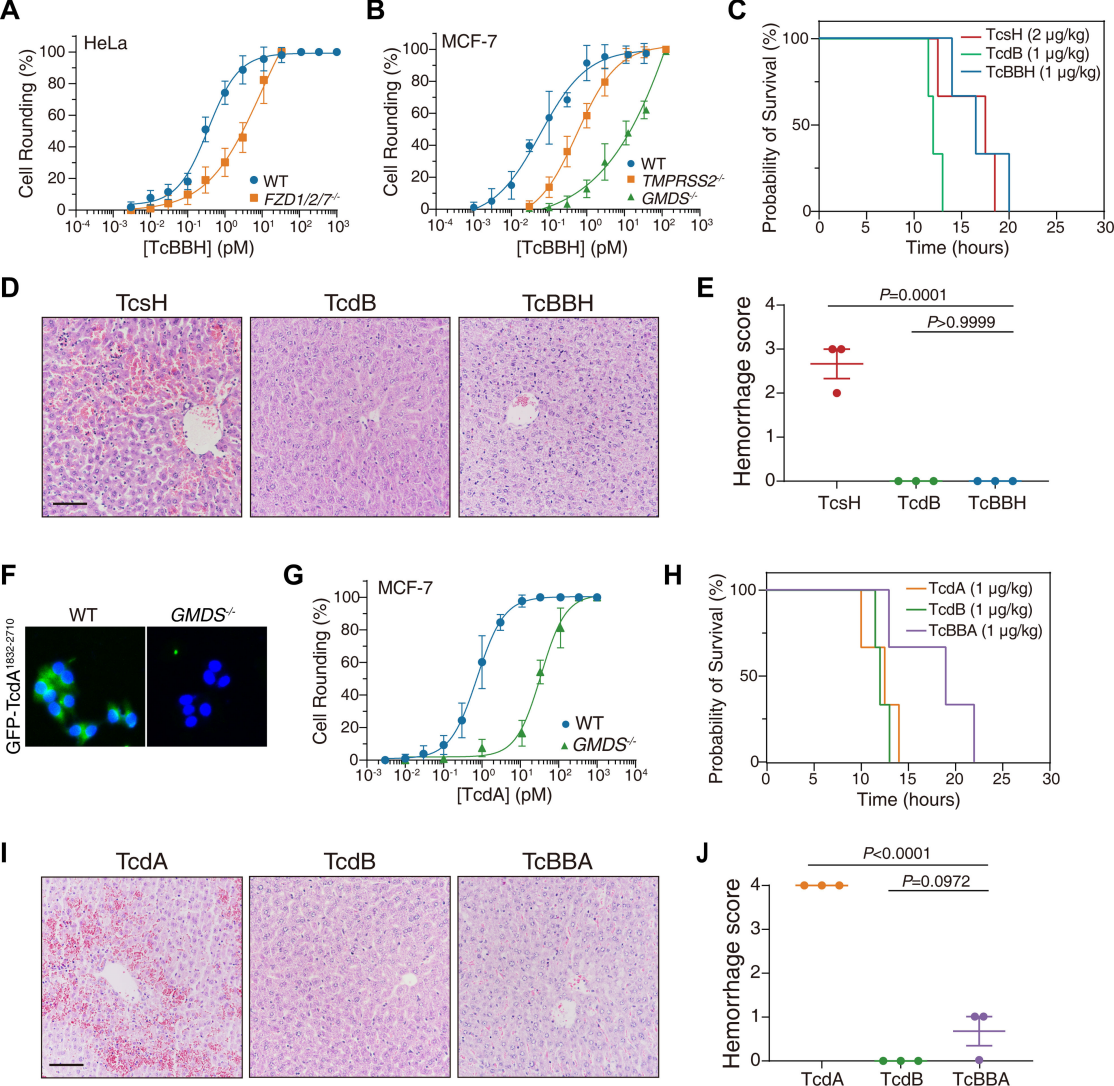
TcdB fused with TcsH or TcdA CROPs cause no hepatic hemorrhage. (**A**) The sensitivities of HeLa and *FZD1/2/7*^‒/‒^ cells to TcBBH were measured using the cytopathic cell-rounding experiments. (**B**) The sensitivities of MCF-7 WT, *TMPRSS2*^‒/‒^, and *GMDS*^‒/‒^ cells to TcBBH were measured using the cytopathic cell-rounding experiments. (**C**) Kaplan-Meier curves show the survival of C57BL/6 WT mice intraperitoneally injected with 2-µg/kg TcsH, 1-µg/kg TcdB, or 1-µg/kg TcBBH, respectively, Log-rank (Mantel-Cox) test. (**D**) Mouse liver tissues were harvested 8 hours post-toxin injection and sectioned for H&E staining histopathology (scale bar, 100 µm). (**E**) Histological scores for the hemorrhagic pathology in panel** D** were assessed. Error bars indicate mean ± SEM, *n* = 3 mice per group, ordinary one-way analysis of variance (ANOVA). (**F**) Representative fluorescence images show GFP-TcdA^1832–2710^ (green) binding to the MCF-7 WT but not *GMDS*^‒/‒^ cells. Cell nuclei were stained by Hoechst (blue), and the scale bar represents 50 µm. (**G**) The sensitivities of MCF-7 and *GMDS*^‒/‒^ cells to TcdA were measured using the cytopathic cell-rounding experiments. (**H**) Kaplan-Meier curves show the survival of C57BL/6 WT mice IP injected with 1-µg/kg TcdA, 1-µg/kg TcdB, or 1-µg/kg TcBBA, respectively. (**I**) Mouse liver tissues were harvested 8 hours post-toxin injection and were sectioned for H&E staining histopathology (scale bar, 100 µm). (**J**) Histological scores for the hemorrhagic pathology in panel **I** were assessed. Error bars indicate mean ± SEM, *n* = 3 mice per group, ordinary one-way ANOVA. For panels **A**, **B**, and **G**, error bars represent the mean ± SD, *n* = 6.

Since the target preference of TcsH is insufficient for hemorrhagic toxicity, we next tried to find clues from receptor selectivity. It was shown that the CROP domain-truncated TcsH does not induce hepatic hemorrhage (17). To investigate whether the CROP-mediated receptor binding can cause hemorrhagic effects, we used the CROP domain of TcsH to replace the homologous part in TcdB. This newly generated chimeric toxin was named TcBBH (Fig. 2A). It has been previously defined that TcdB binds to its receptor frizzled proteins (FZDs) via DRBD (23), and the TcsH CROP domain binds to transmembrane protease serine 2 (TMPRSS2) and fucosylated glycans (FGs) (9, 17). Using cytopathic cell rounding assays, we showed that the HeLa wild-type (WT) cells are more sensitive to TcBBH compared to the *FZD1/2/7*^‒/‒^ cells, indicating TcBBH utilizes FZDs as cellular receptors (Fig. 3A). We also tested the cytotoxicity of TcBBH in the MCF-7 WT, *TMPRSS2*^‒/‒^, and *GMDS*^‒/‒^ cells. GMDS is a cytosolic enzyme that produces GDP-fucose, a substrate for fucosylation. The *GMDS*^‒/‒^ cells were shown to have minimized surface fucosylation (17). As expected, the MCF-7 *TMPRSS2^‒/‒^* and *GMDS^‒^*^/‒^ cells were more resistant to TcBBH compared to the WT cells, indicating TcBBH also uses TMPRSS2 and FGs as cellular receptors (Fig. 3B). When applied in mice, 1-µg/kg IP-injected TcBBH killed the mice in ~12 to 24 hours (Fig. 3C; Fig. S1B). Despite being toxic, TcBBH induced no extravasation of red blood cells in the mouse livers (Fig. 3D and E).

We next tested TcdA, the other hemorrhage-inducible LCT. TcsH and TcdA have no shared protein receptor to our knowledge, but they likely bind to similar sugar moieties via their CROPs based on previous studies (17, 24, 25). Cell surface binding experiment revealed that the CROP domain of TcdA robustly binds to FGs on the cell membrane (Fig. 3F). We further showed that cell surface FGs effectively mediated the cellular entry of TcdA (Fig. 3G; Fig. S2).

We then built a TcdB-TcdA chimeric toxin (TcBBA) by fusing the first 1,900 residues from TcdB and the entire TcdA CROP domain (residues 1,832–2,710 in TcdA) (Fig. 2A). We validated that TcBBA uses FZDs and FGs as cellular receptors (Fig. S3) and is potent in mice (Fig. 3H; Fig. S1C). Similar to TcBBH, IP injection of TcBBA caused minimal erythrocyte extravasation in the mouse liver (Fig. 3I and J). These results suggest that the CROP domains of TcsH and TcdA are not sufficient factors to cause hepatic hemorrhage.

### C-terminally truncated TcsH with partial CROPs could induce hemorrhage

In the previous study, we showed that 2-µg/kg TcsH^1-1832^ failed to kill the mice and induce hepatic hemorrhage (17). With a much higher dose (50 µg/kg), this CROP-less TcsH was able to kill the mice within ~12 to 24 hours (Fig. S4A). Interestingly, 50-µg/kg TcsH^1-1832^ still caused no overt hemorrhagic lesions in the mouse livers (Fig. S4B), indicating that the lack of the CROP domain impairs the hemorrhagic toxicity of TcsH.

**Fig 4:**
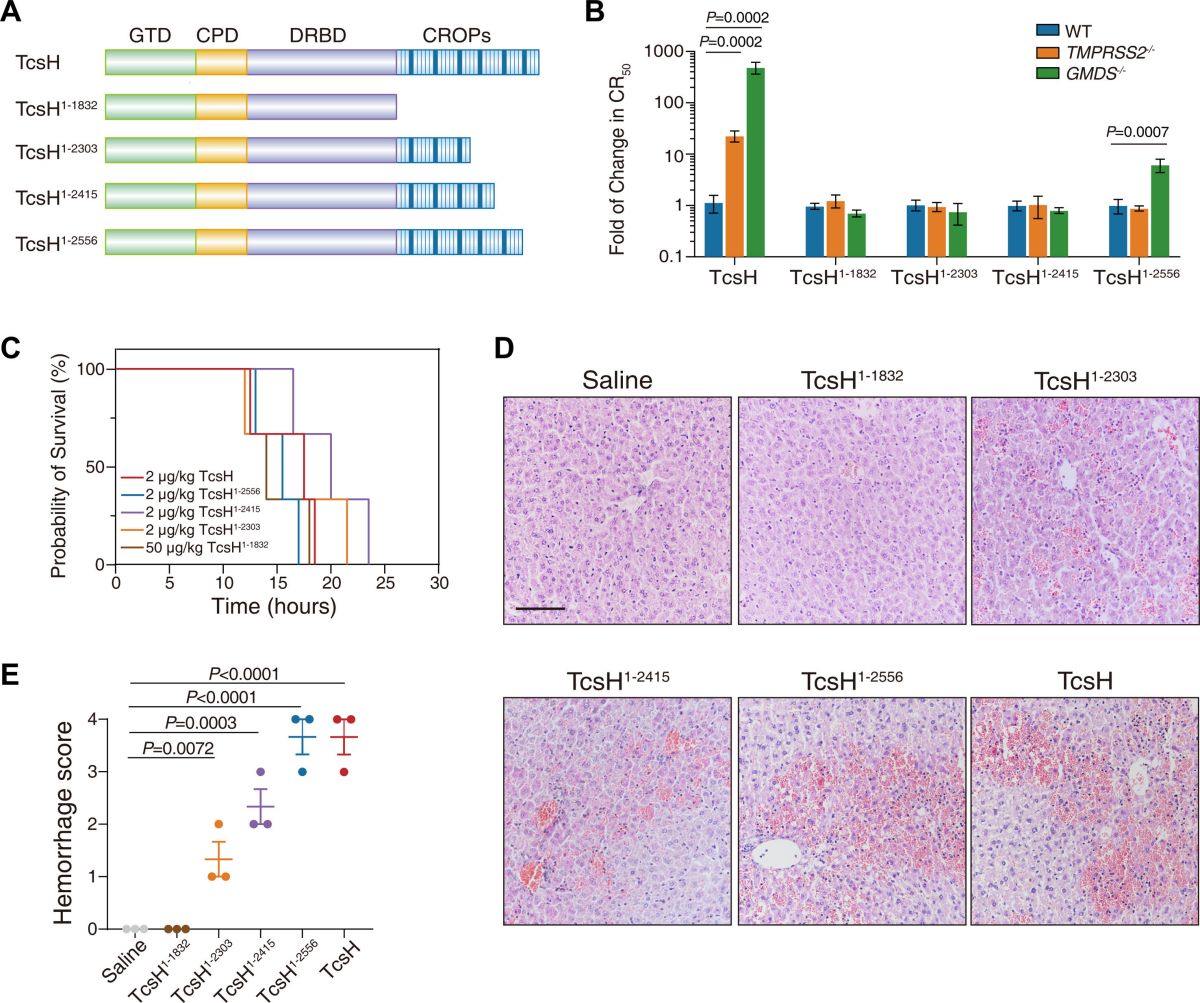
C-terminally truncated TcsH with partial CROPs induced hemorrhage. (**A**) Schematic drawing of C-terminally truncated toxins, including TcsH^1-1832^, TcsH^1-2303^, TcsH^1-2415^, and TcsH^1-2556^. (**B**) The change of toxin resistance in the *TMPRSS2*^‒/‒^ and *GMDS*^‒/‒^ cells were quantified and normalized to the MCF-7 WT. Error bars represent the mean ± SD, *n* = 6. (**C**) Kaplan-Meier curves show the survival of C57BL/6 WT mice IP injected with 50-µg/kg TcsH^1-1832^, 2-µg/kg TcsH^1-2303^, 2-µg/kg TcsH^1-2415^, 2-µg/kg TcsH^1-2556^, or 2-µg/kg TcsH, respectively. (**D**) Mouse liver tissues were harvested 8 hours post-toxin injection and sectioned for H&E staining histopathology (scale bar, 100 µm). (**E**) Histological scores for the hemorrhagic pathology in panel** D** were assessed. Error bars indicate mean ± SEM, *n* = 3 mice per group, ordinary one-way ANOVA.

**Fig 5:**
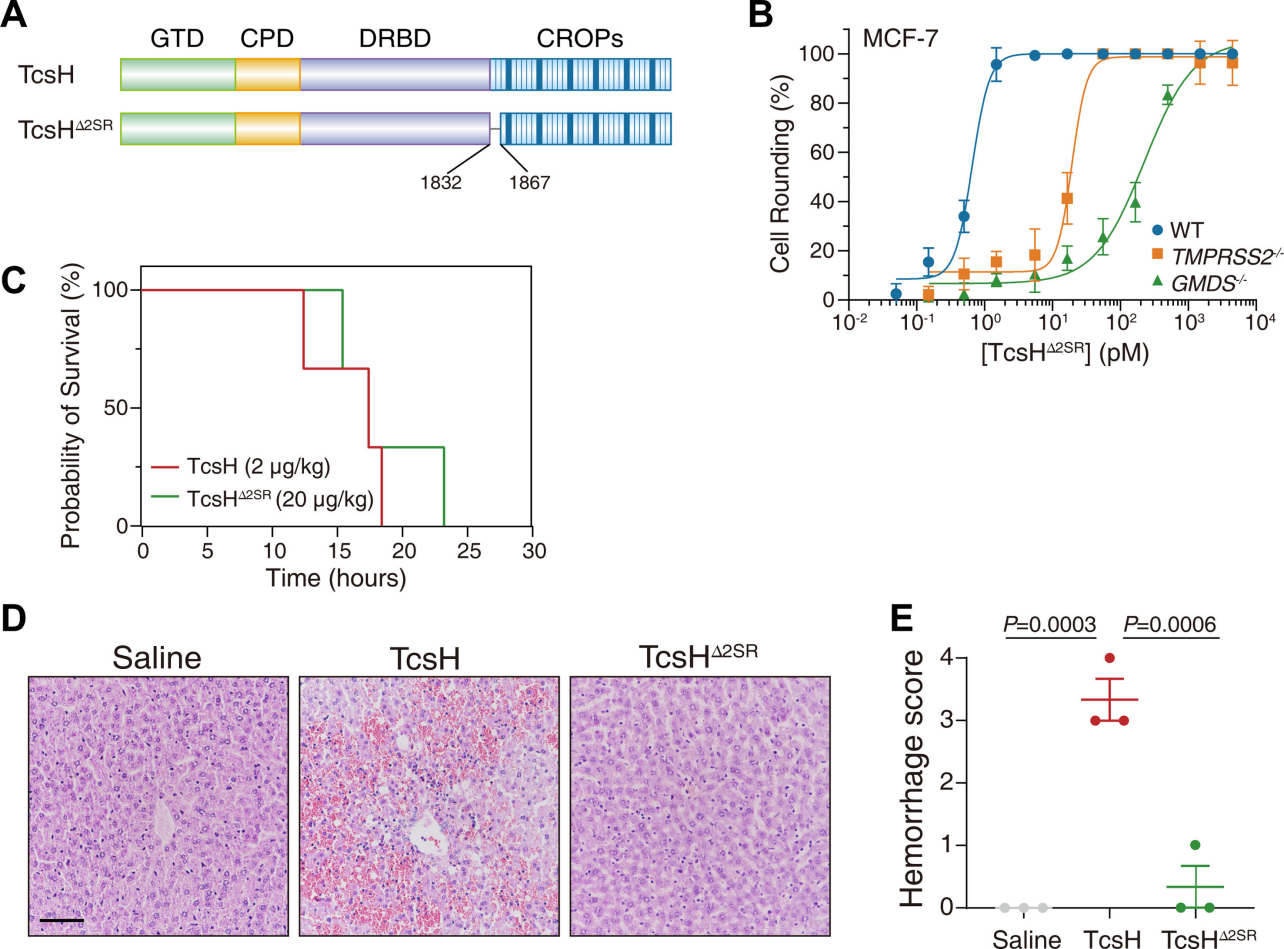
Disruption of the first two SRs in TcsH impairs hemorrhagic toxicity. (**A**) Schematic drawing of TcsH^∆2SR^, an internally deleted TcsH mutant. (**B**) The sensitivities of MCF-7 WT, *TMPRSS2*^‒/‒^, and *GMDS*^‒/‒^ cells to TcsH^∆2SR^ were measured using the cytopathic cell-rounding experiments. Error bars represent the mean ± SD, *n* = 6. (**C**) Kaplan-Meier curves show the survival of C57BL/6 mice IP injected with 2 μg/kg TcsH or 20 μg/kg TcsH^∆2SR^, respectively. (**D**) Mouse liver tissues were harvested 8 hours post-toxin injection and sectioned for H&E staining histopathology (scale bar, 100 µm). (**E**) Histological scores for the hemorrhagic pathology in panel** D** were assessed. Error bars indicate mean ± SEM, *n* = 3 mice per group, ordinary one-way ANOVA.

Therefore, we managed to interrogate the specific segment within the CROP domain that accounts for the hemorrhagic toxicity of TcsH. For this purpose, we further generated some C-terminally truncated TcsH with different lengths of CROPs remaining, including TcsH^1-2556^, TcsH^1-2415^, and TcsH^1-2303^ (Fig. 4A). The intoxication ability of these newly-created truncations, together with TcsH and TcsH^1-1832^, was assessed using the cytopathic cell-rounding assay on the MCF-7 WT, *TMPRSS2*^‒/‒^, and *GMD*^‒/‒^ cells. TcsH^1-2556^ was equally potent on the WT and *TMPRSS2*^‒/‒^ and modestly less toxic (~7-fold) to the *GMD*^‒/‒^ cells, indicating that it partly recognizes FGs but not TMPRSS2. TcsH^1-2415^, TcsH^1-2303^, and TcsH^1-1832^ showed similar potency on three cell lines, suggesting that they could use neither TMPRSS2 nor FGs as cellular receptors (Fig. 4B and 5). Then we challenged the mice with these truncated TcsH to investigate their toxicity *in vivo*. Surprisingly, 2-µg/kg TcsH^1-2556^, TcsH^1-2415^, or TcsH^1-2303^ was sufficient to kill mice within ~12 to 24 hours, which resembled TcsH but not TcsH^1-1832^ (Fig. 4C). Histopathological analysis revealed that TcsH^1-2556^, TcsH^1-2415^, or TcsH^1-2303^ induced severe extravasation of red blood cells in the mouse liver, which was also similar to TcsH but not TcsH^1-1832^ (Fig. 4D and E). These results indicate that the C-terminal half of CROPs is dispensable for hemorrhagic toxicity while necessary for TMPRSS2/fucosylation-mediated cellular entry of TcsH.

### Disruption of the first two SRs in TcsH impairs hemorrhagic toxicity

It was previously reported that the first few oligopeptide repeats played important roles in both receptor-binding and autoprocessing regulation in TcdB (26, 27). Therefore, we next generated a TcsH mutant with the first two oligopeptide repeats disrupted (deleting residues 1,832–1,867) and named it TcsH^∆2SR^. The impact of ∆2SR on cytotoxicity was monitored using the MCF-7 WT, *GMDS*^‒/‒^, and *TMPRSS2*^‒/‒^ cells. TcsH^∆2SR^ is as potent as TcsH in the MCF-7 cells and effectively uses TMPRSS2 and FGs for cellular entry (Fig. 5B).

TcsH^∆2SR^ was then used to challenge the mice via IP injection. This mutant is less potent compared with TcsH in mice, as a dose of 20 µg/kg was required to kill the mice within ~12 to 24 hours (Fig. 5C; Fig. S1D). Strikingly, TcsH^∆2SR^ did not induce notable erythrocyte extravasation in the mouse livers, which is similar to TcsH^1-1832^ (Fig. 5D and E).

### The ∆2SR in TcdA also dissects the hemorrhagic and lethal toxicity

**Fig 6:**
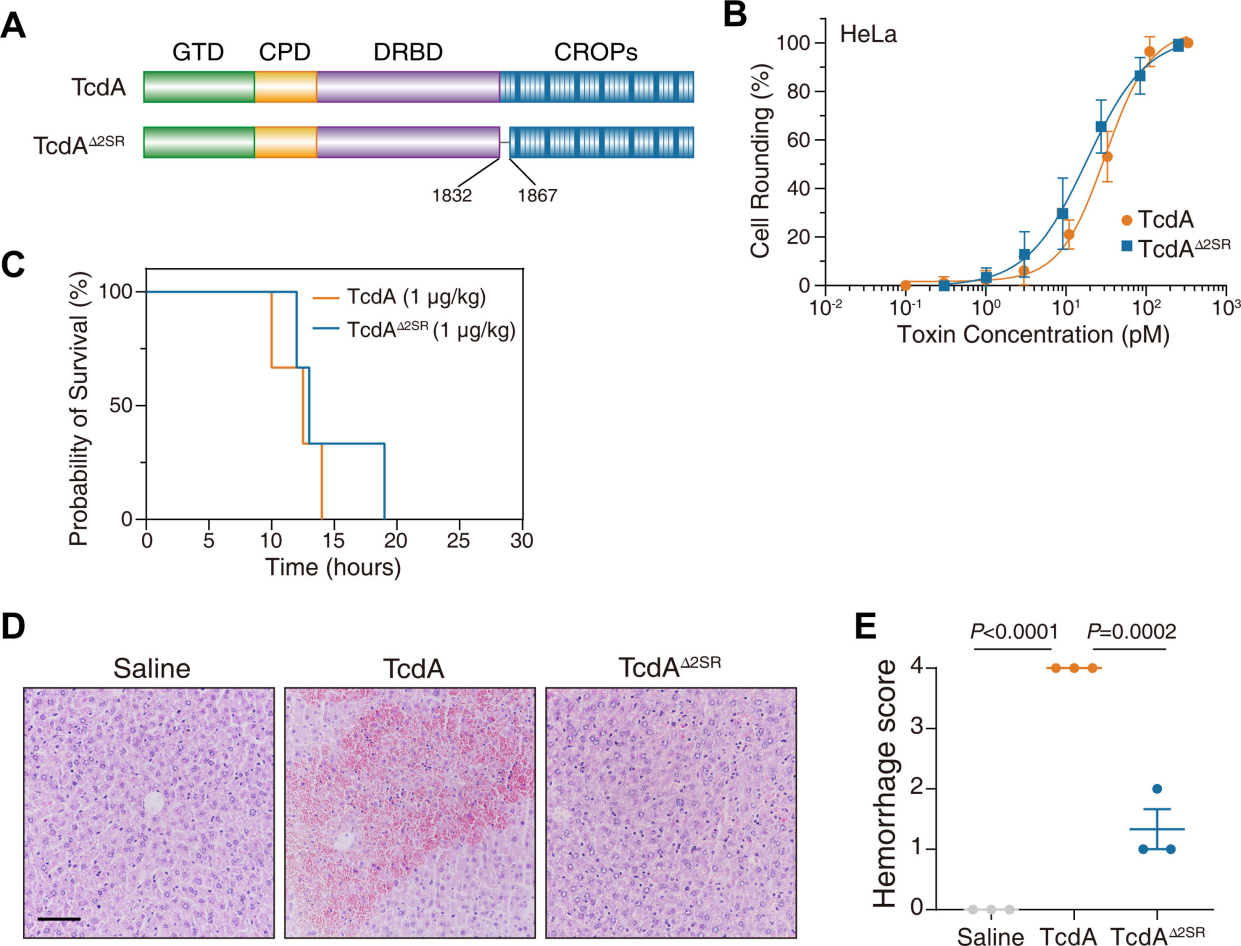
The ∆2SR in TcdA also dissects the hemorrhagic and lethal toxicity. (**A**) Schematic drawing of TcdA^∆2SR^, an internally deleted TcdA mutant. (**B**) The sensitivities of HeLa cells to TcdA and TcdA^∆2SR^ were measured using the cytopathic cell-rounding experiments. Error bars represent the mean ± SD, *n* = 6. (**C**) Kaplan-Meier curves show the survival of C57BL/6 mice IP injected with 1-μg/kg TcdA or 1-μg/kg TcdA^∆2SR^, respectively. (**D**) Mouse liver tissues were harvested 8 hours post-toxin injection and sectioned for H&E staining histopathology (scale bar, 100 µm). (**E**) Histological scores for the hemorrhagic pathology in panel **D **were assessed. Error bars indicate mean ± SEM, *n* = 3 mice per group, ordinary one-way ANOVA.

Since TcdA is sequentially close to TcsH, we also generated a TcdA mutant (TcdA^∆2SR^) with its first two SRs disrupted (Fig. 6A). TcdA^∆2SR^ showed similar toxicity to the full-length TcdA in the HeLa cells, indicating that the internal deletion did not impair the cytotoxicity of TcdA (Fig. 6B).

In the toxin challenge assay, TcdA^∆2SR^ and TcdA showed practically equal lethality in the mice (Fig. 6C). Notably, while TcdA caused massive erythrocyte extravasation in the mouse livers, TcdA^∆2SR^ barely induced hepatic hemorrhage (Fig. 6D and E). The results of all LCT derivatives in the toxin challenge assays are summarized in Table 1.

**TABLE 1:** Summary of the LCTs and their derivatives used in this study^*c*^

Toxin	GTD activity	Cellular receptors	Lethality^*a*^	Hepatic hemorrhage^*b*^
TcsH	TcsH	TMPRSS2 and FGs	High	Yes
TcdA	TcdA	LDLR, LRP1, sGAGs, and FGs	High	Yes
TcdB	TcdB	FZDs, CSPG4, and PVRL3	High	No
TcsL	TcsL	SEMA6A/SEMA6B	High	No
Tcnα	Tcnα	LDLR, LRP1, LRP2, and sGAGs	High	No
TpeL	TpeL	LRP1	Low	No
TcHBB	TcsH	FZDs	High	No
TcBBH	TcdB	FZDs, TMPRSS2, and FGs	High	No
TcBBA	TcdB	FZDs and FGs	High	No
TcHLL	TcsH	SEMA6A/SEMA6B	High	No
TcsH^1-1832^	TcsH	?	Medium	No
TcsH^1-2303^	TcsH	?	High	Yes
TcsH^1-2415^	TcsH	?	High	Yes
TcsH^1-2556^	TcsH	FGs	High	Yes
TcsH^∆2SR^	TcsH	TMPRSS2 and FGs	Medium	No
TcdA^∆2SR^	TcdA	LDLR, LRP1, sGAGs, and FGs	High	No

^*a*^ The lethality was represented as the dose of a toxin that was needed to kill the C57BL/6 mice within 12–24 hours. High: <10 μg/kg, medium: 10–300 μg/kg, low: >300 μg/kg.

^*b*^ Hepatic hemorrhage was monitored eight hours post-toxin IP injection by histopathological analysis.

^*c*^ PVRL3, poliovirus receptor-like 3; SEMA6A/SEMA6B, semaphorin 6A and 6B; sGAG, sulfated glycosaminoglycan.

## DISCUSSION

TcsH and TcdA are distinct from other LCTs, not only because they harbor the longest CROP domains but also because they have a remarkable ability to induce extensive hemorrhage *in vivo*. In this study, we used IP injection to deliver the toxins into the mice and confirmed that only TcsH and TcdA induced hepatic hemorrhage. Several interpretations were proposed for similar pathological phenomena, such as epithelial barrier disruption, excessive inflammation, and abnormal vascular permeability (28, 29). The question is while these effects could be generally caused by other LCTs, why are TcsH and TcdA special in causing hemorrhage?

In the case of exotoxins, specificities of target and receptor targeting are usually key factors in determining the pathological manifestations (30–32). Because all LCTs glucosylate Rho/Ras family proteins, target specificity is unlikely a reason for the unique hemorrhagic toxicity of TcsH and TcdA. Particularly, TcsH and TcdA shared a very similar spectrum of the target proteins with TcdB, another LCT with minimal hemorrhagic toxicity (20, 21). To further solidify this point, we showed that a chimeric toxin containing TcsH GTD and the remaining parts from TcdB did not induce extensive hepatic bleeding in mice.

Recent studies demonstrated that LCTs recognize highly diverse host receptors, including sulfated glycosaminoglycans (sGAGs), low-density lipoprotein receptor (LDLR), and low-density lipoprotein receptor-related protein 1 (LRP1) for TcdA (33, 34); chondroitin sulfate proteoglycan 4 (CSPG4), poliovirus receptor-like 3 (PVRL3), FZDs, and tissue factor pathway inhibitor (TFPI) for TcdB (35–39); semaphorin 6A and 6B for TcsL (40, 41); FGs and TMPRSS2 for TcsH (17); sGAGs, LDLR, LRP1, and megalin for Tcnα (42, 43); and LRP1 for TpeL (44). These encouraging advancements in identifying LCT receptors have opened possibilities for exploring whether the hemorrhagic toxicity is attributed to specific receptor targeting. Both TcdA and Tcnα recognize sGAGs, LDLR, and LRP1 for cellular entry, but Tcnα induces few bleedings. Thus, these receptors are unlikely factors for causing severe hemorrhage. Both TcsH and TcdA bind to FGs via their CROP domains. However, chimeric toxins containing the CROP domain of TcsH or TcdA failed to induce hepatic hemorrhage, although they are potent in mice. Therefore, binding to FGs is not sufficient for LCTs to cause erythrocyte extravasation in mice.

We then studied the toxicity of TcsH deletion mutants in both cells and mice. Two C-terminal truncated TcsH mutants, TcsH^1-2415^ and TcsH^1-2303^, lose the ability to recognize FGs and TMPRSS2, thus showing drastically reduced cytotoxicity to the MCF-7 cells compared to the full-length TcsH. On the contrary, TcsH^1-2415^ and TcsH^1-2303^ are only slightly less lethal to mice compared to TcsH, and both can induce massive bleeding. This is also in line with our recent findings that TcsH still induced severe hemorrhage in the *TMPRSS2*^‒/‒^ mice. Therefore, the cytotoxicity (in MCF-7) is not always consistent with the lethal and hemorrhagic toxicity of TcsH, while FGs and TMPRSS2 are not necessary receptors to cause hemorrhage *in vivo*.

TcsH^∆2SR^ turns out to be a very intriguing mutant of TcsH. TcsH^∆2SR^ retains the capability of using FGs and TMPRSS2 as cellular receptors, which resembles TcsH. TcsH^∆2SR^ loses the ability to induce hemorrhage, though it is also less lethal compared to the full-length TcsH in mice. We further generated a TcdA mutant (TcdA^∆2SR^) in a similar way. TcdA and TcdA^∆2SR^ are almost equally potent in MCF-7 cells and mice, while only TcdA but not TcdA^∆2SR^ induced extensive erythrocyte extravasation *in vivo*. It is particularly interesting to note that the lethal toxicity and hemorrhagic toxicity of TcsH and TcdA can be dissected. Moreover, the disruption of the first two SRs in both TcsH and TcdA resulted in the loss of hemorrhagic toxicity, implying that both toxins employ a shared mechanism to elicit hemorrhage.

We conjecture that ∆2SR in TcdA and TcsH would affect the recognition of a yet unknown factor, which leads to the hemorrhagic effect *in vivo*. This could potentially be a cell surface receptor, an intracellular pattern recognition receptor, or a signaling regulator. Recent studies demonstrated that the homologous segment in TcdB is required for CSPG4 binding. More precisely, CSPG4 binds to a region where CPD, DRBD, and CROPs converged (30, 45). Given the high structural similarity among core parts of LCTs (9, 46–49), the undefined factor(s) may bind to TcsH and TcdA in a resembled manner.

Lastly, our findings reinforce the notion that LCTs may elicit complex pathological effects through multiple host receptors/factors/pathways. For instance, TcdB utilizes four types of receptors including FZD, TFPI, CSPG4, and PVRL3. FZD and TFPI are alternative epithelial receptors for different TcdB subtypes that mainly contribute to epithelial disruption and inflammation, whereas CSPG4-mediated entry causes strong edema in the gut (23, 37, 38, 45, 50). For TcsH, TMPRSS2-mediated cellular entry largely contributes to the epithelial disruption; thus, the hemorrhagic effect is likely due to another undefined host factor or signaling pathway. Taken together, this study could help us understand the action mechanisms of TcsH and TcdA as well as the pathogenesis of *C. difficile* and *P. sordellii* infections. It also provides important clues for elucidating the underlying mechanisms of toxin-induced hemorrhagic pathology *in vivo*.

## MATERIALS AND METHODS

### Materials

The following reagents and recombinant protein were purchased from the indicated vendors: ClonExpress MultiS One Step Cloning Kit (C113, Vazyme), 2× Phanta Flash Master Mix (P510-01, Vazyme), FastPure Gel DNA Extraction Mini Kit (DC301-01, Vazyme), AxyPrep Plasmid Miniprep Kit (AP-MN-P-250, AxyPrep), 2× MultiF Seamless Assembly Mix (RK21020, Abclonal), hematoxylin solution according to Delafield (03971, Sigma-Aldrich), Eosin Y solution (318906, Sigma-Aldrich), and recombinant Tcnα (TX20111-50, HZZC Technology Co., Ltd.).

### Cell lines

HeLa (H1, CRL-1958) and MCF-7 (HTB-22) cells were originally obtained from the American Type Culture Collection. They tested negative for mycoplasma contamination and were authenticated via STR profiling (Shanghai Biowing Biotechnology Co. Ltd, Shanghai, China). MCF-7 *GMDS*^‒/‒^, MCF-7 *TMPRSS2*^‒/‒^, and HeLa *FZD1/2/7*^‒/‒^ cells were previous laboratory stocks (17, 30). All cells were cultured in Dulbecco's modified eagle medium supplemented with 10% fetal bovine serum and 0.1-mg/mL streptomycin/penicillin, in a humidified atmosphere of 95% air and 5% CO_2_ at 37°C.

### Mice

C57BL/6 mice (male and female, 6–8 weeks) were purchased from the Laboratory Animal Resources Center at Westlake University (Hangzhou, China). Mice were housed in specific pathogen-free micro-isolator cages with free access to drinking water and food, monitored under the care of full-time staff. All mice had a 12-hour cycle of light/darkness (7 a.m.–7 p.m.), housed at 20°C–24°C with 40%–60% humidity.

### Creating truncated and chimeric toxin constructs

Gene fragments encoding TcdA (reference sequence: *C. difficile* R20291), TcsH (reference sequence: *P. sordellii* 9048), TcsH^1-1832^, TcsH^1-2303^, TcsH^1-2415^, and TcsH^1-2556^ were PCR amplified and cloned into the pHT01 vector with an additional C-terminal His-tag. To construct the chimeric toxins TcHBB and TcHLL, the gene fragment residues 1–520 from TcsH were fused to the gene fragment residues 522–2,366 from TcdB or residues 521–2,364 from TcsL before insertion into the pHT01 vector, respectively. For chimeric toxin TcBBH, residues 1–1,834 from TcdB were combined with residues 1,832–2,618 from TcsH and together were cloned into the pHT01 vector. Likewise, the chimeric toxin TcBBA was created by combining DNA encoding residues 1–1,900 of TcdB with the gene encoding residues 1,832–2,710 of TcdA. For obtaining two mutant toxins, TcsH^∆2SR^ and TcdA^∆2SR^, gene residues 1,868–2,618 of TcsH or residues 1,868–2,710 of TcdA were fused to residues 1–1,831 from TcsH or TcdA, respectively, which were devoid of their first two oligopeptide repeats. All constructs were validated by DNA sequencing.

### Expression and purification of recombinant proteins

Recombinant TcdA, TcdB, TcsL, TcsH, TpeL, TcsH^1-1832^, TcsH^1-2303^, TcsH^1-2415^, TcsH^1-2556^, and chimeric proteins were expressed in *Bacillus subtilis* SL401 and purified as His-tagged proteins (18). In brief, *B. subtilis* cells were cultured at 37°C until OD_600_ reached 0.6 and then were induced with 1-mM isopropyl-β-D-thiogalactoside at 25°C for 24 h. Proteins were purified by Ni-affinity chromatography, followed by Superdex-200 (GE Healthcare) size-exclusion chromatography.

### The cytopathic cell rounding assay

The cytopathic effect of LCT-derived toxins was analyzed using the gold-standard cell-rounding assay. Briefly, cells were exposed to toxins for 12–14 hours. The phase-contrast images of the cells were captured by a microscope (Olympus IX73, ×10 or ×20 objectives) with the software Olympus CellsSens Standard (v.2.1). Six zones of 300 μm × 300 µm were selected randomly, with each zone containing 20–50 cells. Round-shaped and normal-shaped cells were counted manually. The percentage of round-shaped cells was analyzed using GraphPad Prism (v.9.0.0; GraphPad Software, LLC).

### Toxin challenge assays in mice

C57BL/6 mice (6–8 weeks old, male and female) were intraperitoneally injected with different toxins, respectively. The animals were monitored for up to 5 days post-challenge for toxic effects and mortality. Survival was graphed as Kaplan-Meier curves. For histopathological studies, the mice were injected with the toxins and then euthanized after 8 hours. The livers were excised out, fixed, paraffin-embedded, sectioned, and subjected to H&E staining.

### H&E staining and histopathological analysis

Liver specimens were fixed in a 4% formaldehyde aqueous solution for 12 hours before dehydration with gradient alcohol. The samples were then cleared with xylene and embedded in paraffin. Paraffin blocks were cut into 5-µm sections and stained by H&E. The H&E liver staining sections were scored based on hemorrhage on a scale of 0–4 (mild to severe). The average scores were plotted on the charts.

### Statistics and reproducibility

Data are presented as mean ± standard deviation. The number of the sample size (*n*) and the statistical hypothesis testing method (ordinary one-way analysis of variance) are described in the legends of the corresponding figures. Statistical analyses of data were performed with GraphPad Prism (v.9.0). An asterisk denotes a *P* value of <0.05, and n.s. means without statistical significance.

## Data Availability

All data that support the findings of this study are available from the corresponding authors upon reasonable request. Source data are provided in the paper.

## SUPPLEMENTAL MATERIAL

Supplemental figures spectrum.00354-24-s0001.pdf Fig. S1-S5.

ASM does not own the copyrights to Supplemental Material that may be linked to, or accessed through, an article. The authors have granted ASM a non-exclusive, world-wide license to publish the Supplemental Material files. Please contact the corresponding author directly for reuse.
