# Supplementary material for: Identification of a hemorrhagic determinant in Clostridioides difficile TcdA and Paeniclostridium sordellii TcsH
Source: Microbiol Spectr. 2024 May 6;12(6):e00354-24. doi: 10.1128/spectrum.00354-24 (PMC11237598; doi:10.1128/spectrum.00354-24)
Supplement: Supplemental figures — Fig. S1-S5. [file spectrum.00354-24-s0001.pdf]

Supplementary Figures

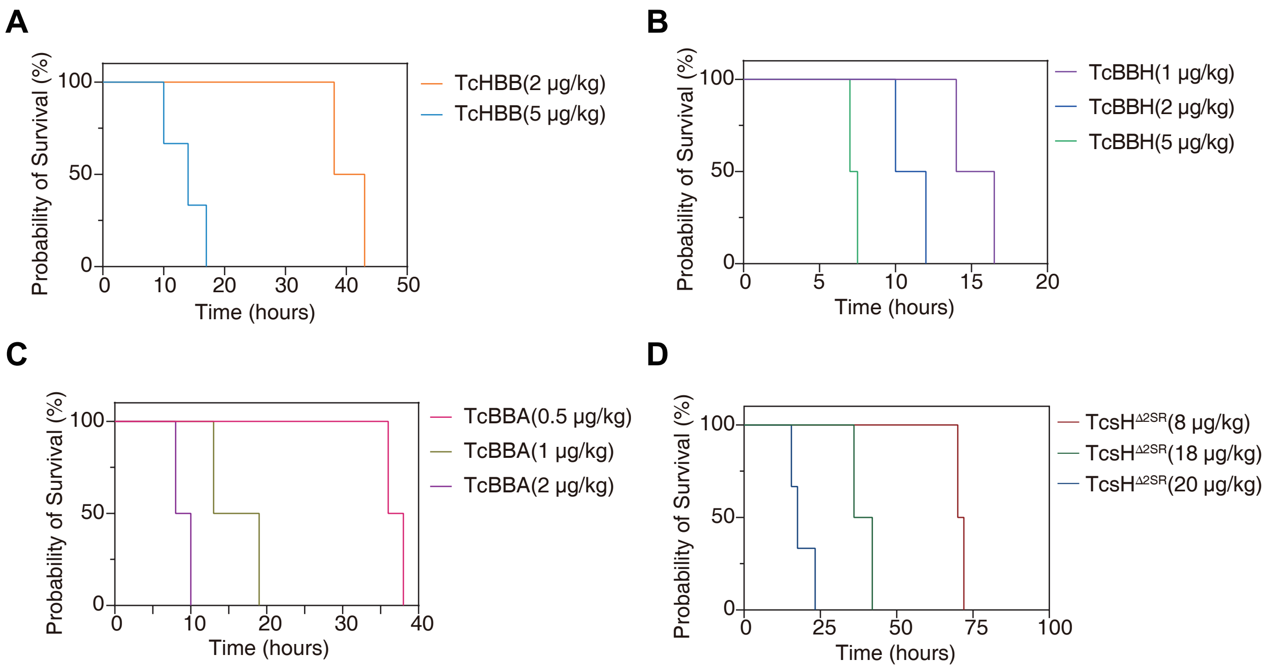

S1 Fig. Survival curves of the mice IP-injected with different dosages of TcHBB, TcBBH, TcBBA, and TcsH$^{\Delta 2SR}$.

(A) Kaplan-Meier curves show the survival of the mice IP-injected with 2 μg/kg or 5 μg/kg TcHBB, respectively. (B) Kaplan-Meier survival curves of the mice IP-injected with 1, 2, or 5 μg/kg TcBBH, respectively. (C) Kaplan-Meier survival curves of the mice IP-injected with 0.5, 1, or 2 μ g/kg TcBBA, respectively. (D) Kaplan-Meier survival curves of the mice IP-injected with 8, 18, or 20 μg/kg TcsH$^{\Delta 2SR}$, respectively.

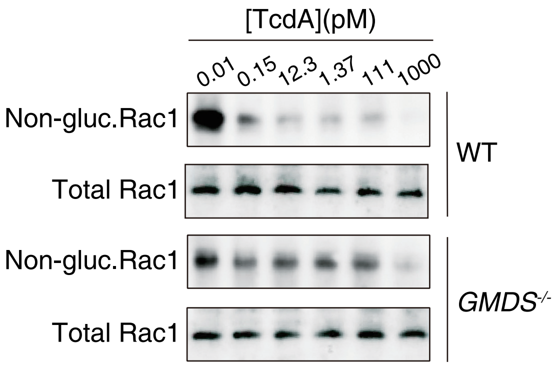

S2 Fig. RAC1 glucosylation of MCF-7 WT and *GMDS*$^{-/-}$ cells post exposed to TcdA. The sensitivities of MCF-7 WT and *GMDS*$^{-/-}$ cells to TcdA were compared by analyzing the glucosylation level of RAC1 via immunoblot assays.

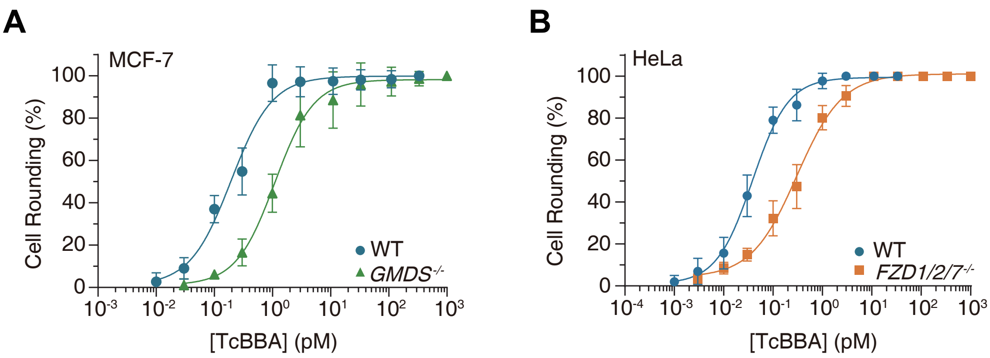

S3 Fig. TcBBA uses FZDs and FGs as cellular receptors.
(A) The sensitivities of MCF-7 and *GMDS*$^{-/-}$ cells to TcBBA were measured using the cytopathic cell-rounding experiments. (B) The sensitivities of HeLa and *FZD1/2/7*$^{-/-}$ cells to TcBBA were measured using the cytopathic cell-rounding experiments. Error bars represent the mean ± s.d., n = 6.

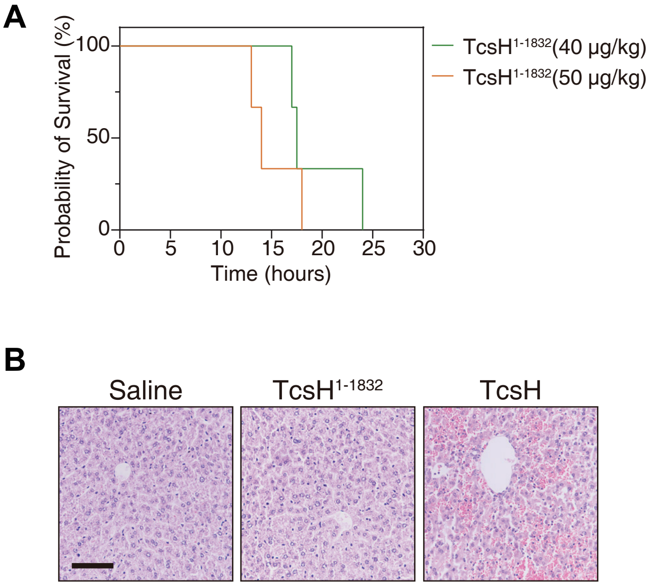

S4 Fig. TcsH$^{1\text{-}1832}$ caused no hepatic hemorrhage in mice.
(A) Kaplan-Meier curves show the survival of the mice IP-injected with 40 or 50 µg/kg TcsH$^{1\text{-}1832}$, respectively. (B) Mouse livers were harvested 8 hours post-toxin injection and sectioned for H&E staining histopathology (Scale bar, 100 µm.).

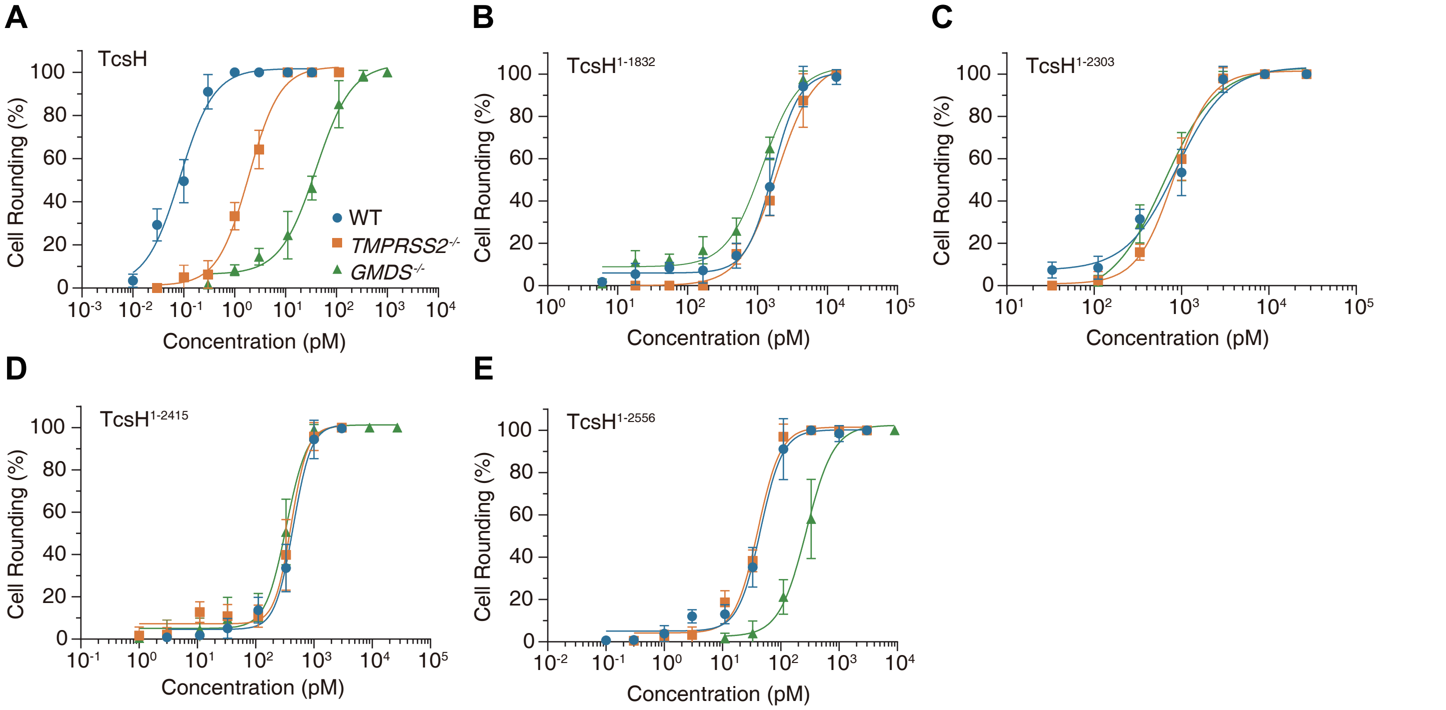

S5 Fig. C-terminally truncated TcsH failed to recognize TMPRSS2 and FGs.
(A-E) The sensitivities of MCF-7 WT, *TMPRSS2*$^{-/-}$, and *GMDS*$^{-/-}$ cells to TcsH (A), TcsH$^{1-1832}$ (B), TcsH$^{1-2303}$ (C), TcsH$^{1-2415}$ (D), and TcsH$^{1-2556}$ (E) were measured using the cytopathic cell-rounding experiments. (Error bars represent the mean ± s.d., n = 6.).
